# A Case of Brucellosis Presenting with Multiple Hypodense Splenic Lesions and Bilateral Pleural Effusions

**DOI:** 10.1155/2011/614546

**Published:** 2011-05-25

**Authors:** Emine Dilek Eruz, Serhat Birengel, Alpay Azap, Gulden Yilmaz Bozkurt

**Affiliations:** Department of Clinical Microbiology and Infectious Diseases, Faculty of Medicine, Ankara University, 06100 Ankara, Turkey

## Abstract

Brucellosis is a zoonotic infectious disease, which mainly present with lymphoreticular system invovement. However any organ system can be attacked by the microorganism. In this paper we present a 52-year-old female patient who was admitted to the Infectious Diseases Department with complaints of fatigue, arthralgias, fever, and weight loss. In the medical examination and radiological analysis bilateral pleural effusions and hepatosplenomegaly were detected. Serum transaminase levels were two times higher than the upper limits of normal. Abdominal ultrasound revealed sludge in the gallbladder and multiple hypodense splenic lesions (the largest was 1 cm in diameter). Brucella melitensis was isolated from the blood culture of the patient. Rifampicin (600 mg/day) and doxycycline (200 mg/day) therapy was started. Follow-up chest radiography and ultrasonography revealed the absence of pleural effusion. Splenic lesions and hepatosplenomegaly were totally regressed. The patient has been followed for 3 months after 6 week antibiotic regimen without recurrence. Brucellosis was expected to be the cause of all pathological signs.

## 1. Introduction

Brucellosis is an endemic zoonosis in Turkey. Fever, night sweats, fatigue, myalgia, and arthralgias are common symptoms. Hepatomegaly and splenomegaly are frequent findings on physical examination [1]. Rarely, brucellosis can cause abscess formation in the spleen, liver, or any other organs. Empyema, pleural effusion, interstitial pneumonia, pulmonary granuloma formation, solitary pulmonary nodules, hilar and mediastinal lymphadenopathy or pneumothorax can be seen as a result of brucella bacteremia [2]. In this paper we present a case of brucellosis with multiple organ system involvement.

## 2. Case Presentation

A 52-year-old female patient was referred to the Infectious Diseases Department of the Faculty of Medicine Ankara University with complaints of fatigue, arthralgias, fever, and 10 kg weight loss over two months before admission. At the time of hospitalization, her vital parameters were body temperature: 38.0°C, blood pressure: 100/80mm/Hg, heart rate: 88 bpm, and respiratory rate: 22 bpm. Lung auscultation revealed decreased respiratory sounds in both lower lobes. Chest radiography showed bilateral pleural effusions and diffuse infiltrative opacities in the bases of both lungs. Her physical examination showed a palpable mass in the liver 4 cm below the right costal margin, along the midclavicular line. The spleen was not palpable but with percussion splenomegaly could be determined. No abnormal physical examination finding was present in the other systems. On laboratory findings serum transaminase levels were two times higher than the upper limits of normal; she had a mild leucopenia and an elevated CRP level (see Table 1). After blood cultures were performed from both arms, empiric 2 g ceftriaxone twice daily intravenously and clarithromycin 1 gm twice daily orally was started because of a suspected respiratory tract infection. In the following days the patient's fever had subsided. Abdominal ultrasound revealed hepatosplenomegaly, sludge in the gallbladder, intrahepatic venous congestion, and multiple hypodense splenic lesions (the largest one was 1 cm in diameter). Thoracic CT scan showed bilateral pleural effusions and atelectasis in adjacent lung zones. A subpleural nodular opacity was detected in the right apical segment, and peribronchovascular interstitial thickening was seen in the right lower lobe. On the basis of these findings, ultrasound-guided thoracentesis was planned, but the patient refused any invasive procedure. 

*Brucella melitensis* was isolated from the blood cultures obtained at the time of hospitalization, on the fourth day of incubation. The antibiotic therapy was switched from ceftriaxone and clarithromycin to oral rifampicin 600 mg/day and doxycycline 200 mg/day. The Brucella isolate was sent to a reference laboratory, and it was reported as *Brucella melitensis* biotype 3. The titers of brucellosis antibodies were measured as 1 : 640 (seropositive) using the standard tube agglutination test. The patient had no history of contact with animals, consuming fresh cheese or milk, but she had a history of eating raw meatballs (a traditional Turkish food which is made with raw meat and pounded wheat) two months before admission.

The patient was reevaluated for a suspected brucella endocarditis, but her cardiac examination and transthoracic echocardiography was normal. Because she also had a complaint of mild back pain, thoracolumbar MRI was performed without pathology seen. 

Fifteen days after the initial course of brucellosis treatment, no pleural effusion or pulmonary consolidation was seen on the follow-up chest radiography and ultrasonography. At the end of the 24th day abdominal ultrasonography was repeated. Splenic lesions and hepatosplenomegaly were totally regressed, and a normal gallbladder was seen. Brucellosis was expected to be the cause of all pathological signs that were previously seen in the lungs and abdomen, and the regression of these was assumed as a response to the therapy. 

The patient was discharged in good health after a 32-day hospital stay. She completed a 6-week drug regimen at home. During this time all her complaints have resolved. She has been followed for 3 months after the end of therapy without recurrence.

## 3. Discussion

Brucellosis may present with many different symptoms. A broad spectrum of clinical manifestations can be seen, including multisystem involvement or asymptomatic infection [3]. Therefore, diagnosis can be difficult. Fever, night sweats, fatigue, myalgia, and arthralgias are common symptoms. Brucellosis can cause complications such as spondylitis, sacroiliitis, osteomyelitis, endocarditis, epididymo-orchitis, meningitis, and encephalitis. Anemia, leucopenia, and thrombocytopenia can be seen due to bone marrow involvement. Localized or generalized lymphadenopathy may be detected [1]. Also brucellosis may induce microangiopathic hemolytic anemia, thrombotic thrombocytopenic purpura, and hemolytic uremic syndrome [3]. Unusually, vasculitis can be seen secondary to skin involvement [4]. Renal involvement is rare but can result in acute renal failure [5]. During the course of brucellosis, even disseminated intravascular coagulation (DIC) and shock may develop [6].

Hepatosplenomegaly can be detected in physical examination, and blood chemistry may reveal elevated transaminases. Brucellosis can lead to nonspecific hepatic inflammation, granulomatous hepatitis, and rarely to cirrhosis [7, 8]. Many different histologic patterns can be seen in hepatic involvement, but the most common histological appearance is granuloma formation in the liver parenchyma and in the periportal areas [8]. Brucella bacteremia can cause abscess formation in the spleen, liver, or in other organs. Even soft tissue abscesses can occur [9]. However, the development of isolated splenic abscess is extremely rare [10]. During the course of brucellosis, splenic subcapsular hematoma also may develop. In a few rare cases, spontaneous splenic rupture due to brucella infection has been reported [11]. The incidence of splenic abscess in patients with acute brucellosis is low and does not exceed 2-3% of the cases, even in the largest case series [12, 13]. In 2006, Pourbagher et al. reviewed 251 cases of brucellosis from Turkey and identified pleural effusion in 2.8%, splenic abscess in 1.6%, splenic cyst in 0.8%, and acute acalculous cholecystitis in 0.4% of the cases with an ultrasound. Excepting a few cases with acute appendicitis, acute cholecystitis, and multiple splenic cysts, all of the patients were treated with combinations of either two or three antibiotics without any need for surgery [14]. In the study of Colmenero et al. hepatosplenic abscesses were detected in 7 (0.8%) of 805 patients. The ratio of hepatic to splenic abscesses were identified as 57.1% and 42.9%, respectively. Therapeutic failure was defined as the lack of regression in the lesions after appropriate antibacterial treatment for two months. In 5 patients (0.6%) surgical treatment (splenectomy or surgical drainage) was needed [12]. In our case, abdominal ultrasound revealed hepatosplenomegaly, sludge in the gallbladder and multiple hypodense splenic lesions, suggestive of splenic abscess. Twenty-four days after the initial course of brucellosis treatment, abdominal ultrasound was normal.

Respiratory system involvement in brucellosis is very rare. Empyema, pleural effusion, interstitial pneumonia, pulmonary granuloma formation, solitary pulmonary nodules, hilar and mediastinal lymphadenopathy or pneumothorax can be seen as a result of brucella bacteremia [2, 15–20]. In a variety of studies of brucellosis, respiratory system involvement has been identified in 1–5% of the cases [2, 15]. Some patients may have complaints of hoarseness or rarely hemopurulent sputum. Although pathological findings in chest radiography are relatively more common, in more than half of these patients no respiratory symptoms can be determined. Common findings are perihilar and peribronchial infiltrations and solitary granulomas [15]. Between 1997 and 1999, Namiduru et al. studied 120 cases of brucellosis from Turkey and identified pulmonary involvement in 2.5% of cases [17]. In the study of Kokoglu et al. pulmonary involvements were detected in 2.1% of 138 cases of brucellosis [18]. Hatipoglu et al. identified 11 (10%) pulmonary involvements of 110 cases of brucellosis. After 6 weeks of drug therapy, clinical and radiological findings were regressed completely in all of the patients, except 4 patients who had COPD and dyspnea [19]. Pappas et al. studied 450 cases of brucellosis between 1999 and 2002 and detected pathological respiratory system findings in 31 (6.9%) of patients involving 3 patients who had pleural effusion. All of these findings were resolved after 6 weeks of drug therapy [2]. In the study of Kochar et al. respiratory system involvement was detected in 7 of 98 cases of brucellosis. A treatment of rifampicin 600 mg/day plus doxycycline 200 mg/day for 6 weeks was given to the patients. After 6 weeks pathological signs on chest radiography persisted in 2 patients, and their treatment was prolonged to 10 weeks. At the end of the therapy, clinical and radiological findings of all patients were improved except in one patient who had calcific opacities on chest radiography. These patients had been followed up for 1 year, and none of them had shown signs of relapse. Respiratory function tests of all patients returned to normal [20]. In the chest radiograph of our patient performed at the time of hospitalization, there were small pleural effusions and diffuse infiltrative opacities in the bases of both lungs. Thoracic CT scan showed bilateral pleural effusions and atelectasis in adjacent lung compartments. Fifteen days after the initial course of brucellosis treatment, no pleural effusion or pulmonary consolidation was seen on the chest radiograph and abdominal ultrasound.

## 4. Conclusion

Brucellosis should be kept in the differential diagnosis of patients who have complaints such as arthralgias, fever, and fatigue. Raw meat, fresh cheese, and milk ingestion are common exposures. Brucella bacteria can play a role in the etiology of pleural effusion and hepatosplenic lesions which may otherwise suggest a malignancy or tuberculosis.

## Figures and Tables

**Table 1:** The laboratory findings obtained at the day of hospitalization and on the seventh day of the rifampicin plus doxycycline treatment.

	AST (IU/L)	ALT (IU/L)	WBC (/mm^3^)	Hemoglobin (g/dL)	Hematocrit (%)	CRP (mg/lt)
Before treatment	64	52	3570	9.0	31.0	13.2
Seventh day of the treatment	22	18	5770	9.5	32.4	1.91
